# Deployment of a Smart Handwashing Station in a School Setting During the COVID-19 Pandemic: Field Study

**DOI:** 10.2196/22305

**Published:** 2020-10-19

**Authors:** Jeremy Herbert, Caitlin Horsham, Helen Ford, Alexander Wall, Elke Hacker

**Affiliations:** 1 Designworks Group Pty Ltd Brisbane Australia; 2 School of Public Health and Social Work Queensland University of Technology Brisbane Australia

**Keywords:** COVID-19, hand hygiene, health promotion, public health, preventive medicine, digital health technology

## Abstract

**Background:**

Hand hygiene is one of the most effective ways to remove germs, prevent the spread of infectious pathogens, and avoid getting sick. Since the COVID-19 pandemic began, health authorities have been advocating good hand hygiene practices.

**Objective:**

The primary aim of this study is to field test a prototype smart handwashing station deployed in a school setting during the COVID-19 pandemic.

**Methods:**

We deployed a smart handwashing station and examined key technological considerations including connectivity, security, and data management systems, as well as the health and safety of users.

**Results:**

The smart handwashing station was deployed for 10 days in a school setting in Australia during the COVID-19 pandemic. The smart handwashing station’s electrical components remained operational during field testing and underwent robust cleaning protocols each day. The handwashing station was used 1138 times during the field test and there was no COVID-19 transmission at the school during the testing.

**Conclusions:**

This study demonstrates that a personalized feedback approach using technology can successfully be implemented at a school and can provide a platform to improve hand hygiene among school-aged children.

## Introduction

COVID-19, a contagious infectious disease caused by SARS-CoV-2, has emerged as a global health crisis and pandemic [1]. To stop the spread and transmission of this virus, behavioral interventions are needed across the population [1]. Hand hygiene is a critical public health control mechanism to prevent the spread of infectious pathogens as the most common way many communicable diseases are transmitted is via hands. Hand hygiene refers to hand cleansing, including washing hands with water and antimicrobial or nonantimicrobial soap, or applying an alcohol-based hand sanitizer to the hands. A meta-analysis suggests improved hand hygiene interventions may reduce rates of gastrointestinal illness by 31% and respiratory illness by 21% [2]. Hand hygiene interventions have been shown to be cost-effective [3]; however, hand hygiene is often not sufficiently practiced, with studies reporting compliance rates between 40% and 60% in both the community and among health care workers [4-6].

Research has shown that COVID-19 can remain on environmental dry surfaces [7]; when a person touches the contaminated surface, they risk infection. Previous research into other coronaviruses (such as SARS-CoV) found that the virus can survive on environmental dry surfaces up to 6 days [8]. Hand hygiene is critically important because even if a person comes into contact with a contaminated surface with their hands, they can avoid becoming infected by washing their hands before they touch their face. Face touching is common, with reports of medical students touching their faces 23 times per hour, 44% of which involved contact with mucous membranes (most commonly the mouth, followed by the nose and eyes) [9]. During the COVID-19 pandemic, germs may be spread more abundantly, with growing evidence suggesting it may be common to have no COVID-19 symptoms [10,11], resulting in these asymptomatic people unknowingly transmitting the virus to others. This is different from previous epidemics such as SARS, where most infected people were symptomatic. During COVID-19, strict social distancing guidelines have been enforced to restrict the spread of the disease in Australia and overseas. However, when restrictions are relaxed and normal routines resume, the flattened epidemic curve may rise again. After the initial outbreaks, COVID-19 may be expected to reoccur in waves, such as seasonally during winter [12]. Although vaccines are in development, we do not know their protective efficacy, nor the number of individuals willing to be vaccinated. Protective behavior such as good hand hygiene among our community will be a critical factor in the control of COVID-19.

Previous studies have shown that health education on hand hygiene alone is often ineffective at changing hygiene behaviors. Current hand hygiene education, such as visual signage, is generic and may not be personalized or unique to the individual. Increasingly, posters are made available at sinks to support the public in effective hand hygiene technique. Previously, hand hygiene posters showed multiple steps and therefore may have been difficult to follow. Recently, a simpler 3-step hand hygiene technique was found to improve technique and compliance compared to a 6-step technique [13]. However, posters that show the technique alone are the least effective for behavioral change, whereas multimodal technologies are much more effective [14]. An intervention using posters placed in university restrooms showed they had limited effect on hand hygiene [15]. Posters tend to act as reminders, and performance feedback is required to encourage optimal technique to ensure all areas of the hands are cleaned. The emotion of disgust has been shown to be a useful intervention strategy in promoting hand hygiene [16]; places considered dirty or with visible dirt proved to be strong hand-hygiene triggers [17]. Other determinants associated with successful hand-hygiene interventions include comfort, social norms, and habit [18].

There are limitations to the current methods of measuring hand hygiene compliance and performance. The World Health Organization (WHO) guidelines recommend that hand hygiene compliance should be assessed by direct observation by trained observers as the gold standard. Direct observation is currently recommended because it is the only approach that can detect all hand hygiene opportunities and actions to assess the number of times hand hygiene was required as well as the appropriate timing of handwashing [19]. Hand hygiene can also be assessed by self-report via interviews or questionnaires; however, self-assessment data are typically overestimated and do not correlate highly with compliance measured by direct observation [19]. Current technologies to measure compliance include electronic counting systems (ie, devices that count how often the soap or sanitizer bottles were used), video monitoring, and automated monitoring systems [20]. The Internet of Things (IoT) is a concept where ordinary items are upgraded to include connectivity, allowing them to transmit information without requiring human interaction.

IoT-enabled smart devices were used in a hospital to measure how often hospital workers washed their hands; sensors monitored the flow of people and tracked disinfectant usage [21]. A limitation of compliance monitoring is that it does not measure hand hygiene performance, which can be analyzed using microbiological tests to assess bacterial counts. These include swab-based sampling for cultures, or finger imprints pressed onto an agar plate [22,23]. These require microbiological expertise and may involve dilutions and laboratory processing, which are time-consuming and costly [23].

During the COVID-19 pandemic, there has been a call for novel, digital behavioral interventions to facilitate adoption and maintenance of individual-level preventive behaviors [12]. In this study, we developed a smart handwashing station as a performance feedback approach to improving hand hygiene. Performance feedback interventions aim to increase awareness of behaviors and may serve as a motivator to continue to perform well or to improve performance [24]. A field test of the smart handwashing station was undertaken in a school setting. We examined key functionality aspects as well as implementation and regulatory considerations.

## Methods

### Smart Handwashing Station Development

In this study, we developed a smart handwashing station, incorporating a 365 nm UV light emitting diode (LED) light source, digital camera, and processing electronics within housing constructed from plastic and mounted on a stand. A commercially available pressure sensor mat (Radio Parts Pty Ltd) was connected via cable to a custom data acquisition system that reported pressure information in real time over a USB connection. Both the pressure sensor mat and the handwashing station were connected to a tablet (Acer Incorporated) via a USB cable. A software application written in Python triggered the camera to take a photo each time a person stood on the pressure sensor mat, and then every 5 seconds that they remained standing on the mat. All images were captured with timestamp information. Data was only stored locally on the tablet, and was periodically collected and sent to an off-site location. Although the device was supervised in this installation, it could easily be fixed to a specific location using a laptop-style antitheft lock.

### Observational and Safety Testing

To check the smart handwashing station was connected and recording usage data correctly, observational testing was performed in Brisbane, Australia. Three volunteers (90 kg, 65 kg, and 30 kg) stood on the pressure sensor mat and used the smart handwashing station 10 times. The time-stamped data collected by the station was then compared with observational data.

The temperature of the smart handwashing station following 2 hours of continuous operation was recorded using an infrared handheld thermometer (ThermaTwin TN410LCE Infrared Thermometer). The UV radiation emitted by the UV light source was measured using a UV intensity meter (Solar Light Co, Model PMA2100) fitted with a digital sensor (Solar Light Co, Model PMA2101). The detector head of the sensor was positioned with consideration of where a person’s hands would be placed during use.

### Smart Handwashing Station Deployment

The field study was conducted during the COVID-19 pandemic in April and May 2020, when handwashing was required every day. One smart handwashing station was deployed to a school located in Queensland, Australia. Children and adolescents aged 5-18 years attended the school, with over 1500 students enrolled. The smart handwashing station was placed near the school entry and outside handwashing facilities in a high-traffic area accessed by all students at the start of the day and during breaks. A moisturizer that contains a UV-fluorescent compound (BREVIS Corporation) was dispensed using a touchless automatic system (Décor House).

Feedback was obtained from end users, including teachers and teaching assistants, to design and refine the device. Questions regarding logistics were asked (eg, battery requirements, transport, and if the image quality was sufficient). In addition, email and phone contact details of the researchers were displayed and provided to the teachers for complaints, technical issues, or further information during the deployment. The deployment of the smart handwashing station was to assess device functionality and not human subjects research; therefore, we obtained an institutional ethics review board exemption from the Human Research Ethics Committee of the Queensland University of Technology.

### Statistical Analysis

Observational testing of the handwashing station dichotomized usage variables to categorical data: handwashing usage data was coded “yes” if timestamped data was recorded to the tablet and coded “no” if the handwashing station was used but no data was recorded to the tablet. The Cohen κ score was calculated to determine if there was agreement between categorical variables for handwashing usage. Values of 0.4-0.6 were considered moderate agreement, values of 0.6-0.8 were considered substantial agreement, and values of 0.8-1.0 were considered almost perfect agreement. GraphPad Prism (Version 8, GraphPad Software Inc) and SPSS (Version 25.0, IBM Corp) were used for analyses.

## Results

### Smart Handwashing Station Operational Function and Safety Testing

**Figure 1:**
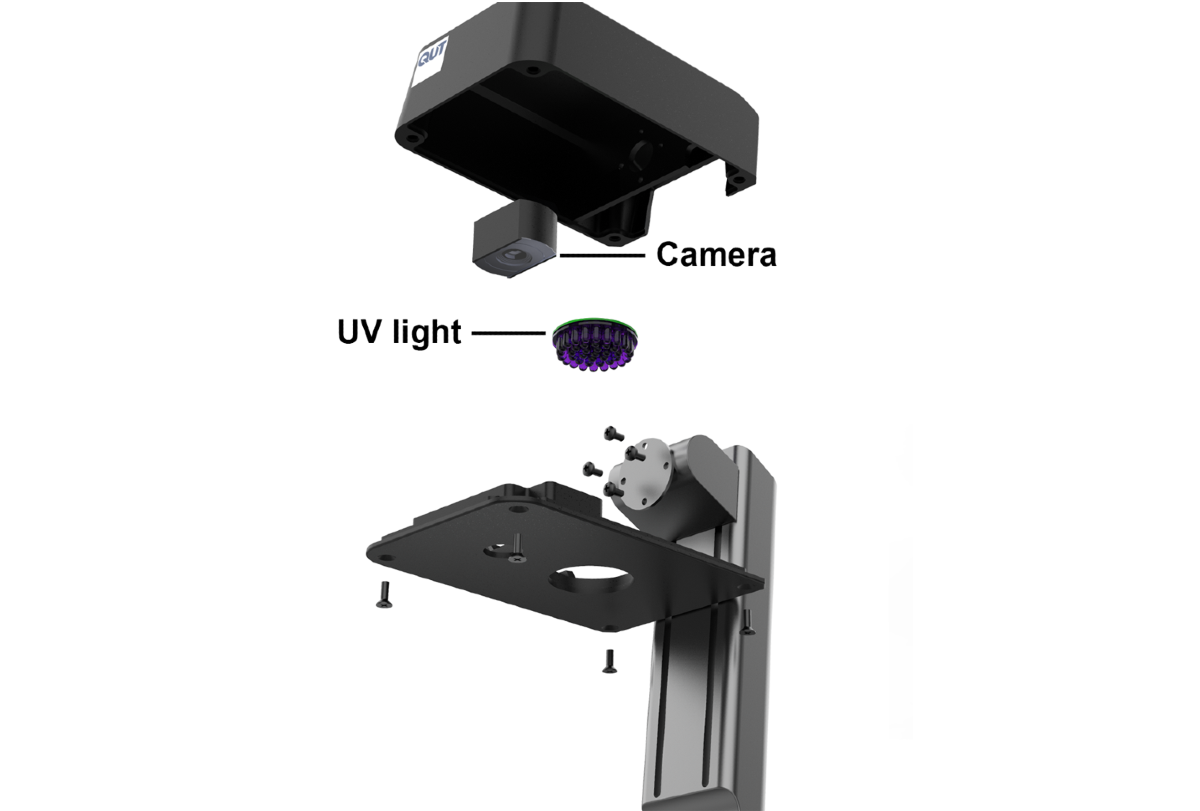
Smart handwashing station with electrical components housed within the top box.

**Figure 2:**
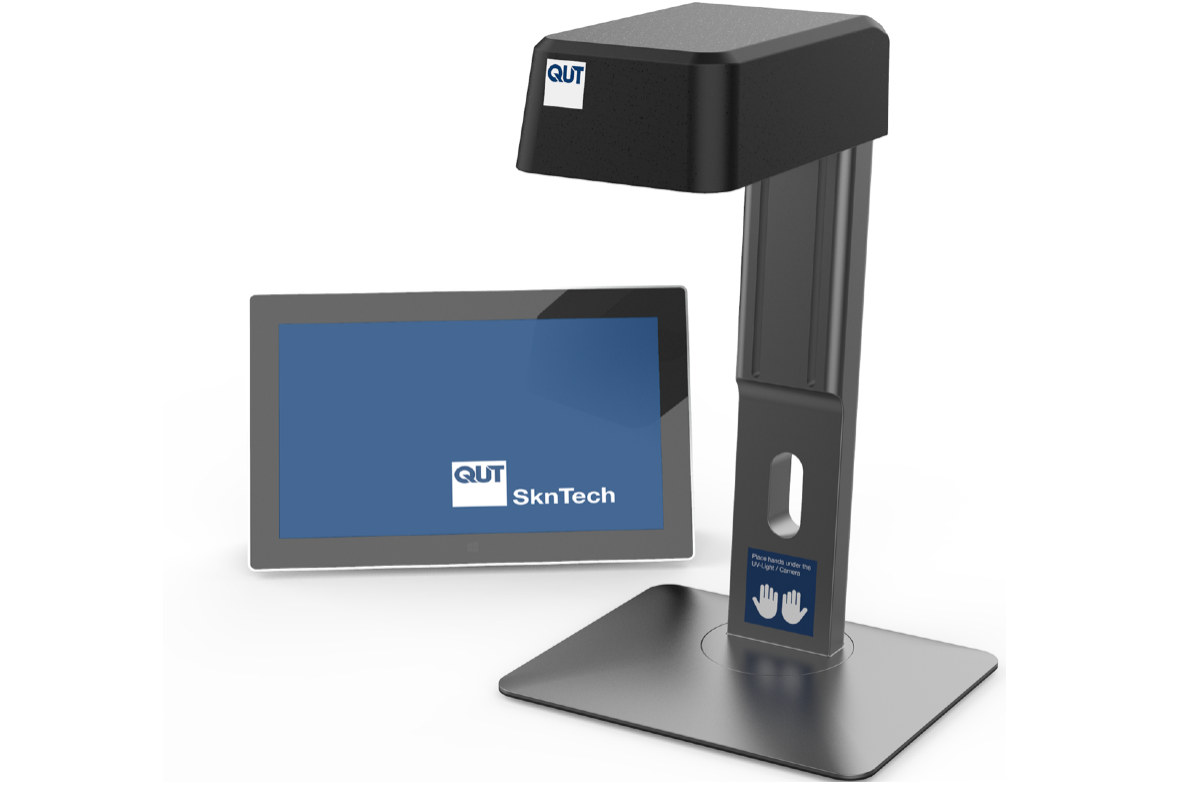
The smart handwashing station was mounted on a stand with a tablet connected to display images.

A prototype smart handwashing station was developed, which uses a UV light source and digital camera to provide personalized feedback on hand hygiene (Figure 1). First, the user applies a moisturizer that contains a UV-fluorescent compound. Second, the user washes their hands and checks their hands at the station (Multimedia Appendix 1). Any areas highlighted have not been washed enough to remove the moisturizer, indicating missed areas that require further washing. This visualization tool provides personalized feedback about where improvements can be made for each user (Figure 2).

The smart handwashing station integrated IoT approaches to track usage and collect images of users’ hands. Observational testing of the smart handwashing station demonstrated there was perfect agreement with observed handwashing and station-recorded usage (κ=1.0, 95% CI 1.00-1.00; Table 1).

The prototype unit emits only UV-A radiation. Over 7 hours of continuous exposure would be needed to equal the level of UV-A received in 15 minutes from the summer sun in Brisbane, Australia. During testing, no skin irritation was reported or observed after participants used the station or the fluorescent moisturizer. The temperature of the smart handwashing station following 2 hours of continuous operation was 28.1 °C on the top of the housing and 25.3 °C on the underside, where the light source is emitted.

**Table 1:** Agreement between observed handwashing usage and station-recorded measurements.

Weight of person	Station-reported usage (N=10)	
	Yes, n (%)	No, n (%)
90 kg	10 (100)	0 (0)
65 kg	10 (100)	0 (0)
30 kg	10 (100)	0 (0)

### Field Testing of Smart Handwashing Station

**Figure 3:**
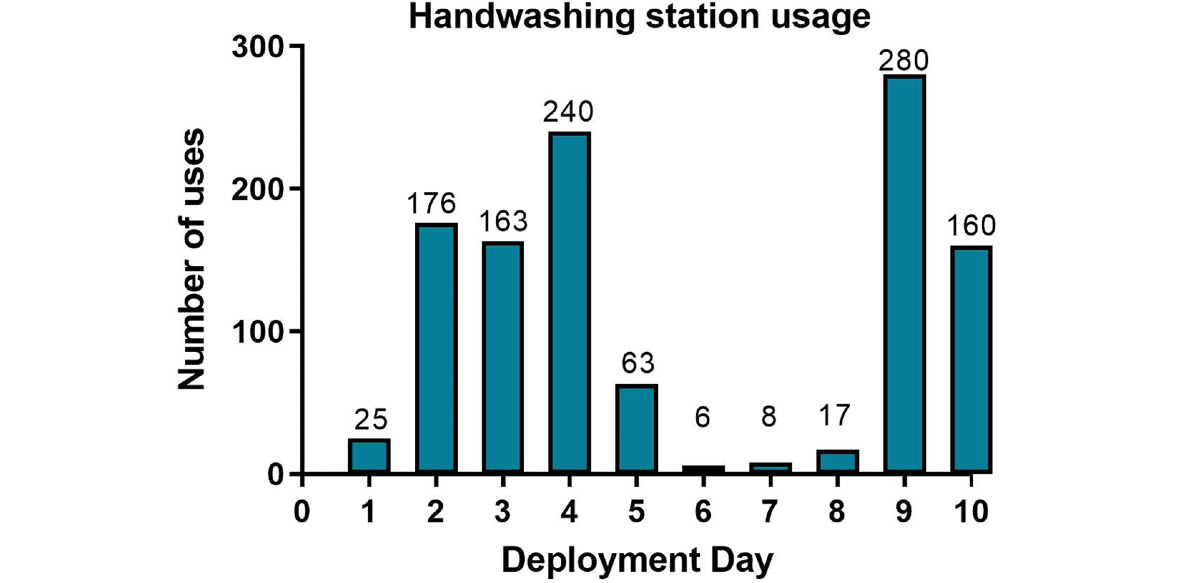
Field testing results of the smart handwashing station. The graph depicts the number of times the smart handwashing station was used each day.

The smart handwashing station recorded data each day during deployment at the school (Figure 3). No complaints, adverse events, or concerns were logged from users or teachers during the 10 days the smart handwashing station was deployed. The handwashing station was stored on a trolley for ease of transport across the school campus and placed outside handwashing facilities in high-traffic areas (Multimedia Appendix 2). The handwashing station was designed so the users did not have to touch or rest their hands on any surface; rather, they could hover their hands between the desk and top of the station. A COVID-19 case was reported at the school several weeks prior to the deployment of the station. During deployment, no transmission of COVID-19 was recorded at the school. The laptop provided sufficient power via the USB connection during field testing. The smart handwashing station was stored in an air-conditioned laboratory room when not in use and the laptop was recharged. The UV light source and digital camera consumed an average of 5 watts of power. As a result, even the relatively small tablet battery was sufficient for over 4 hours of recording before requiring recharging.

## Discussion

Our data demonstrated that the smart handwashing station can provide a visual tool to schoolchildren by highlighting areas on their hands missed during handwashing. The smart handwashing station accurately recorded time-stamped usage data in a school setting in Australia. During the 10-day field test, the smart handwashing station electrical components remained operational and data was received each day, resulting in 100% connectivity. No safety concerns or adverse events occurred when the smart sunscreen station and fluorescent lotion were used by children and adolescents aged 5-18 years. Based on these results, we suggest that the smart handwashing station may become a valuable tool to provide personalized performance feedback on hand hygiene and could help to optimize handwashing techniques in children and adolescents.

Recent advances in technology and wearable sensors have made it possible to measure handwashing performance via automated systems, rather than relying on direct observation with trained observers. Galluzzi et al [25] assessed motion-sensor wristbands that collected data on compliance with the WHO handwashing steps in a clinical setting and found hand hygiene motions could be classified with up to 93% accuracy. However, participants were required to wear the wristbands during handwashing, while the WHO handwashing guidelines recommend removing rings, wristwatches, and bracelets before handwashing; therefore, these wristbands could potentially house infectious pathogens, impeding hand hygiene. Other research assessing the use of hand gestures during handwashing have used armbands placed on forearms and reported recognition rates of 98% and 97%, respectively [26,27]. Armbands increase the mobility of monitoring devices in comparison to stationary devices such as fixed monitoring cameras next to sinks, which have additional privacy concerns due to the collection of identifiable images of people. These wearable sensor trials were also limited by the ability to assess for handedness and its impact on measurements. Most wearable sensor technologies have been developed to measure hand hygiene compliance in health care settings and there may be significant barriers to overcome when implementing this technology in community settings.

This study evaluated the use of a handwashing station in a school environment, which is an ideal setting to introduce health information to young children and teenagers. Children are prone to acquiring acute respiratory illness due to a tendency to put items and their hands in their mouths and noses regularly; such illnesses are transmitted to parents in more than 30% of cases [28]. Studies suggest that modelling handwashing for children, either in-person or by video, when combined with other strategies, can increase correct handwashing [29]. Blacklight technology and UV-sensitive simulated germ lotions have previously been used in school and health care settings and have demonstrated improved handwashing practices using water and soap [30-35] and alcohol-based sanitizers [36], with one study of preschool-aged children reporting a 44% increase in handwashing quality scores [32]. In contrast, Oncu et al [37] reported that the use of blacklight technology and a fluorescent lotion did not increase handwashing quality in primary school students. These previous studies were all conducted prior to the COVID-19 pandemic and future studies exploring behavior change in response to personalized visual feedback may have greater improvements due to the increased awareness of the importance of hand hygiene among the general population.

The smart handwashing station designed during this study was purpose-built to overcome barriers health interventions faced during the pandemic. The smart handwashing station does not require any human interaction during operation and removed the need for a staffing resource during deployment and data collection. Social distancing restrictions were also considered when developing the station, with users able to move from the automatic lotion dispenser to sink areas for washing and then to the station to view feedback in a well-ventilated outdoor space. As it is possible for a person to contract COVID-19 by touching a surface or object that has the virus on it and then touching their own mouth [7], the smart handwashing station was designed as a touch-free platform, with the user’s hands hovering between the desk and top of the station. The station was developed to capture data to provide insights into hand hygiene without being resource intensive; it does not require any infrastructure, such as installed sensors in washrooms. The mobility of the station is also a key design feature; as it is a small unit, it can easily be shipped and deployed in many configurations. The station incorporates IoT capabilities, with time-stamped data collected and images captured. Future work could explore the potential to undertake image analysis on the collected images and generate handwashing quality scores. Technology has the capacity to accelerate and assist public health infectious disease controls and IoT devices may form an essential part of future public health infrastructure and preparedness.

**Figure 4:**
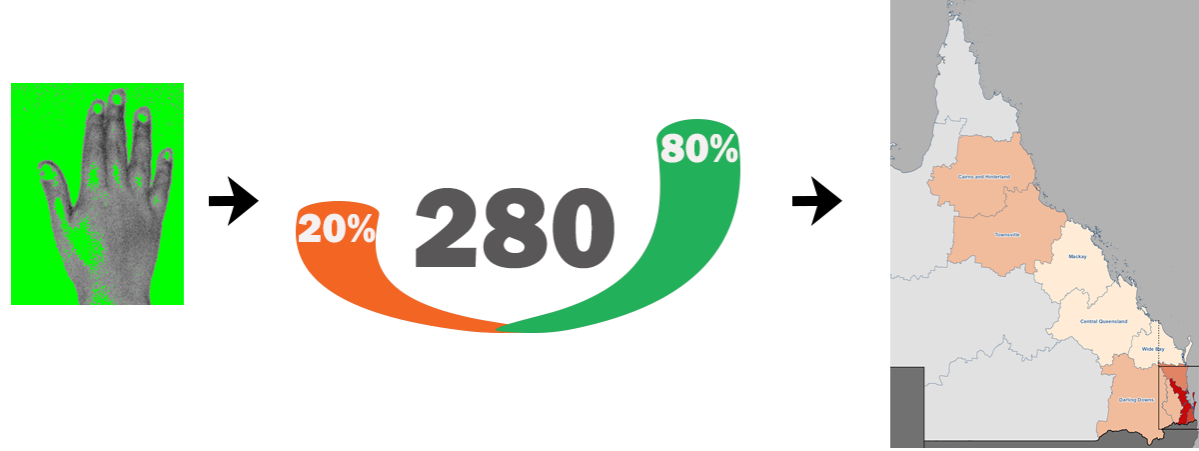
The flow of data from deployed smart handwashing stations to an online dashboard, overlaid with geospatial information. The smart handwashing station could collect data on hand hygiene by analyzing images and determining areas not washed sufficiently (left panel). This information could be used to generate an online dashboard (center panel) indicating the number of times the station was used, as well as the quality of the handwashing, with orange illustrating the frequency of poor handwashing and green representing good handwashing. This information could be overlaid with geospatial data from the location of the handwashing station to indicate areas with poor hand hygiene that have a potentially high risk of transmission (right panel). A graded series of colours could be used to represent risk on a map. The use of pictograms to illustrate the handwashing station data could benefit the translation of information to public health agencies and assist in the delivery of targeted public health programs.

Key technical considerations for a successful deployment included appropriate design for use by school students, with a robust structure, a software system that operates with current organizational platforms, and careful consideration of the configuration to ensure privacy and minimize security risks. Ensuring secure and reliable operation is an important consideration in technology-based deployments, particularly in community areas. The health information gathered by devices during a pandemic can provide potentially critical information to assist public health agencies; however, the information generated needs to be processed in a way that will assist and not overwhelm time-poor health professionals. The flow of information from deployed smart handwashing stations to an online dashboard is shown in Figure 4, illustrating the potential to collect and integrate data to reduce the impact of COVID-19. The use of infographics with overlaid geospatial information can provide insights into potential at-risk populations with low levels of hand hygiene in the community. Delivering targeted prevention programs is essential during a pandemic. With subsequent waves of infections in many countries linked to asymptomatic children and young adults [38,39], interventions that improve hand hygiene in this population are critical. School and college campuses reach high numbers of this target population and this study has shown a smart handwashing station can be deployed in this environment. The economic costs of outbreak-associated lockdowns of schools, travel, and businesses to control the spread of disease are substantial, further highlighting the importance of prevention measures.

The development of new health care devices creates opportunities for skills and knowledge creation through the research process [40]. The smart handwashing station was developed using a stepwise approach, which included first identifying COVID-19 as the target disease and the unmet need of reducing disease transmission by improving hand hygiene. The mechanism by which therapeutic benefits could be derived was then explored and early testing was performed to demonstrate the basic principles of the device. Initial prototyping involved identifying potential challenges in manufacturing, including supply chain delays due to the pandemic. By repurposing computer monitor stands to form the device stand, we were able to overcome potential manufacturing delays and begin preclinical testing. Stakeholder consulting was undertaken and involved discussion of practical elements, such as ease of use and integration into existing practice. This led to determining the school setting as a suitable community site for field testing the prototype device. Schools and colleges have large numbers of children and young adults and the existing education to increase handwashing was in the form of handwashing posters. Comparing the effectiveness of the smart handwashing station’s personalized feedback with printed posters will form part of future research. Before undertaking clinical trials, manufacturing elements including material quality, tooling setup, transport, and shelf-life need to be considered to produce clinical trial prototypes as close to the final device form as possible.

This device would be a low-risk class-1 medical device requiring registration with the Therapeutic Goods Administration (TGA) as a medical device in order to be lawfully sold in Australia. The definition of what constitutes a medical device has recently been updated in Australia by the TGA, with amendments aligning more closely with the equivalent definitions in the European Union’s Regulation (EU) 2017/745. The Federal Drug Administration (FDA) ensures that the claims made by a medical product accurately reflect its risks and benefits in the US market. The regulatory framework determining the medical device approval processes are based on relative risk. Medical devices include a diverse range of devices, from toothbrushes to software. Accelerated approval may be allowed by many regulators if a device can provide evidence that it is substantially similar to an existing device in terms of materials and safety, or if the risk class is sufficiently low as to need relatively little safety data.

While observation is accepted as the gold standard when evaluating handwashing effectiveness, it can be costly and time-consuming, as well as difficult to implement during a pandemic when social distancing and shelter-in-place restrictions are imposed by many governments [41]. Microbiological analysis is another method that can be used to evaluate handwashing effectiveness; however, it requires laboratory processing, which may be impacted during a pandemic as resources are shifted toward screening programs. Microbial flora analysis is a time-consuming process that provides no initial feedback to the user. In addition, the analysis can be impacted by interpersonal variations of permanent skin microflora [23]. The handwashing station in this study included a UV-fluorescent lotion and we assumed that fluorescent-covered parts of the hands would indicate pathogens that remained after washing; however, microbiological validity was not performed in this study.

A limitation of this study is that we did not analyze the usage data or the captured image data to investigate behavior change. In addition, the duration of field testing was only 10 days. Detailed qualitative data was not collected from users. Future research could expand on the enablers and barriers to hand hygiene in schools. In this proof-of-concept study, we did not monitor students’ hand hygiene compliance or handwashing quality. The placement of the station needs to be within close proximity to handwashing facilities and the station was not optimized for hand sanitizer. The Centers for Disease Control and Prevention in the United States recommends using alcohol-based sanitizers only when soap and water are inaccessible. Handwashing using soap and water is more effective for removing most types of microorganisms than using hand sanitizers [42]. Wearing gloves does not replace hand hygiene in the community, as gloves do not provide complete protection against hand contamination and it is not practical for schoolchildren or those in the community to routinely wear gloves.

Future research should explore image analysis approaches to objectively measure hand hygiene levels in the community. Testing satisfaction with the smart handwashing station could be expanded to other settings, including public spaces such as airports, shopping centers, recreational venues, and workplaces. The smart handwashing station shows significant promise for generating relevant data for public health authorities on hand hygiene practices in the community. The smart handwashing station data could be used to inform targeted training as part of health programs in areas with poor handwashing, as well as assist inventory management systems to ensure appropriate levels of soap are always available.

Handwashing is an essential process to reduce the spread of infectious pathogens. This study developed a handwashing station, which used a simple and cheap method that was easily understood by school-aged children. To assist schools to protect their students from outbreaks, the smart handwashing station could help build awareness of the importance of hand hygiene and provide personalized feedback. The usage data generated by this device could also benefit health programs by informing public health authorities of areas with low handwashing where targeted training may increase hand hygiene. This study provides evidence for the technical feasibility of smart handwashing stations in a school setting.

## Appendix

Multimedia Appendix 1 Operational function of the smart handwashing station providing personalized feedback on hand hygiene.

Multimedia Appendix 2 The handwashing station deployed on a trolley outside handwashing facilities in a high-traffic area.

## Abbreviations

IoT: Internet of Things
TGA: Therapeutic Goods Administration
WHO: World Health Organization
