# Supplementary figures and images for: Deployment of a Smart Handwashing Station in a School Setting During the COVID-19 Pandemic: Field Study
Source: JMIR Public Health Surveill. 2020 Oct 19;6(4):e22305. doi: 10.2196/22305 (PMC7575344; doi:10.2196/22305)

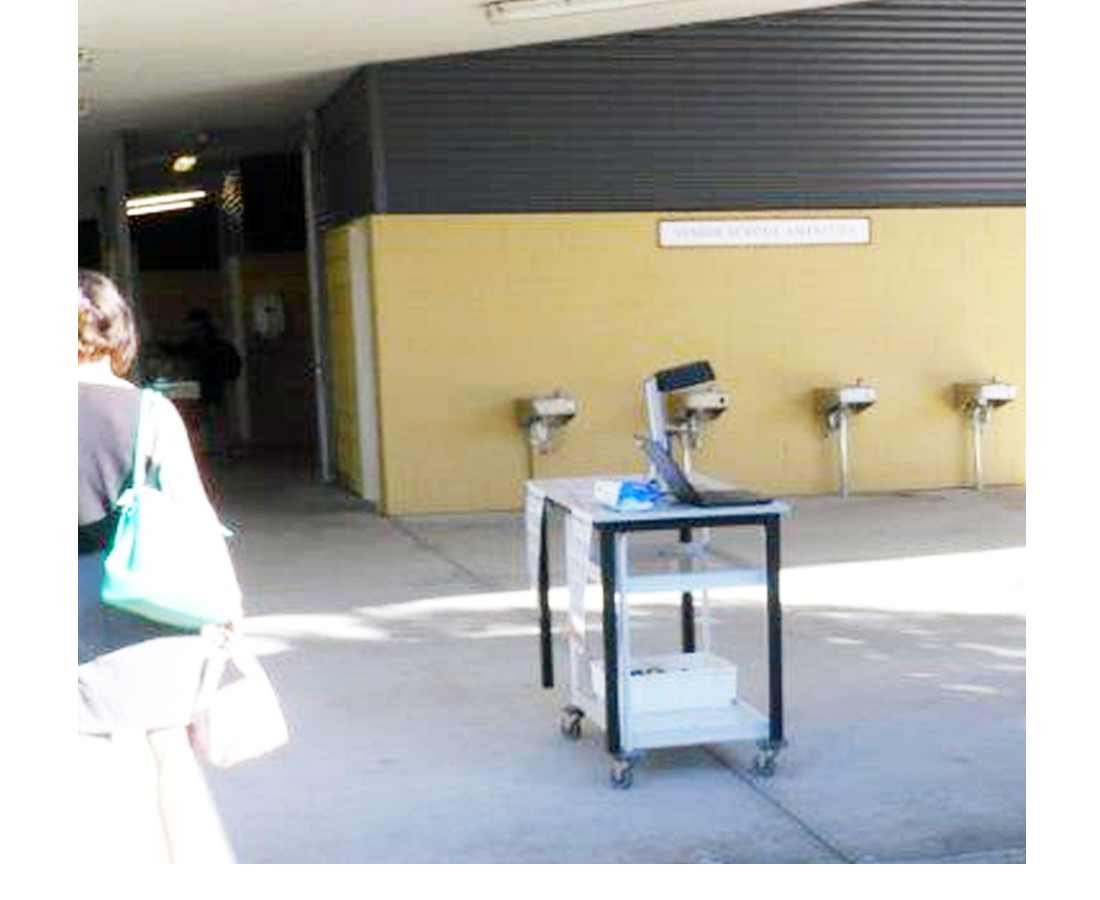

Supplement: Multimedia Appendix 2 [file publichealth_v6i4e22305_app2.png]
